# Molecule-specific interactions of diatomic adsorbates at metal-liquid interfaces

**DOI:** 10.1063/1.4978894

**Published:** 2017-03-17

**Authors:** Jan Philip Kraack, Andres Kaech, Peter Hamm

**Affiliations:** 1 Department of Chemistry, University of Zurich, Winterthurerstrasse 190, CH-8057 Zürich, Switzerland; 2 Center for Microscopy and Image Analysis, University of Zurich, Winterthurerstrasse 190, CH-8057 Zürich, Switzerland

## Abstract

Ultrafast vibrational dynamics of small molecules on platinum (Pt) layers in water are investigated using 2D attenuated total reflectance IR spectroscopy. Isotope combinations of carbon monoxide and cyanide are used to elucidate inter-adsorbate and substrate-adsorbate interactions. Despite observed cross-peaks in the CO spectra, we conclude that the molecules are not vibrationally coupled. Rather, strong substrate-adsorbate interactions evoke rapid (∼2 ps) vibrational relaxation from the adsorbate into the Pt layer, leading to thermal cross-peaks. In the case of CN, vibrational relaxation is significantly slower (∼10 ps) and dominated by adsorbate-solvent interactions, while the coupling to the substrate is negligible.

## I. INTRODUCTION

Molecular properties at solid-liquid interfaces play an outstanding role in physics and chemistry, for instance, in the field of functional materials or in heterogeneous catalysis.^1–5^ A particularly important question relevant to the various fields of surface science is how different molecules at an interface interact with each other, or how they interact with the substrate. This is reasoned by the fact that intermolecular as well as substrate-adsorbate interactions can control the physical and chemical properties of the adsorbates as well as the surface,^6–8^ efficiencies and dynamics of interfacial energy- and charge transfer,^9,10^ or dynamics of surface chemical reactions.^11–13^

For studying molecular properties at surfaces, vibrational spectroscopy has proven its use to yield manifold information.^14–18^ Out of the many methods for vibrational spectroscopy, ultrafast two-dimensional infrared spectroscopy (2D IR) is a powerful tool to observe inter-molecular interactions.^19^ By correlating vibrational signals of an excitation and a detection frequency, the coupling between oscillators^20^ and the dynamics of energy transfer^21^ or chemical exchange^22^ have been resolved with sub-picosecond temporal resolution. 2D IR has been technologically advanced in recent years to also enable investigations of only monolayers of sample molecules at surfaces and interfaces.^16,23–28^ Particularly for adsorbates, the observation of different types of interactions is extremely valuable, since the associated dynamics may be used to extract information about molecular conformations and intermolecular distances under the influence of spatial confinement.^19^

So far, the majority of ultrafast 2D IR studies conducted for various adsorbates on different surfaces has not been able to detect intermolecular interactions via the observation of cross-peaks or vibrational energy transfer.^14,29,30^ Investigations concentrated on coupling between linear- and bridged-bound diatomic adsorbates^31^ or different types of functional groups attached to self-assembled organic monolayers.^14,25,26,29,30^ The only exception are closely packed metal-carbonyl molecules at semiconductor surfaces, for which band splitting and vibrational energy transfer has been observed that were related to aggregation of the sample at the interface.^32,33^ The reason why interactions between adsorbates at interfaces are so hard to observe is largely unresolved to date.

Here, we employ 2D attenuated total reflectance (ATR) IR spectroscopy^16,31,34^ in combination with isotope-labelling of adsorbates at metal-liquid interfaces to investigate intermolecular interactions between diatomic molecules as well as substrate-adsorbate interactions. For this purpose, we employ ^12^C^16^O/^13^C^18^O mixtures of carbon monoxide and ^12^C^14^N^−^/^13^C^15^N^−^ mixtures of cyanide adsorbed to a thin Platinum (Pt) layer. In earlier works on similar sample systems, shifts in the band position of CO on various metal surfaces have been interpreted in terms of strong dipole-dipole coupling that forms vibrational excitons,^35–42^ possibly due to the formation of domains from closely packed CO adsorbates at the interface.^39^ If that interpretation was correct, 2D ATR IR spectroscopy should reveal a direct cross peak at zero waiting time between the vibrational bands of the different isotopomers of the adsorbed molecules. This is, however, not observed, as we will demonstrate. Other related studies investigated the coupling and vibrational lifetimes of adsorbed CO on Pt nanoparticles of controlled size and shape suspended in solution.^43,44^ For very small nanoparticles that still behave like molecules (e.g., 1 nm diameter), the vibrational lifetime was long (∼40 ps) and vibrational energy transfer due to coupling between the CO molecules could indeed be observed, both qualitatively similar to true molecular systems.^20,45^ However, once the properties of the nanoparticle become metallic at sizes ≥2 nm, the vibrational lifetime drops dramatically to ∼2 ps. This observation has been attributed to strong substrate-adsorbate coupling. In this interpretation, the vibrational energy of the adsorbate is transferred to electronic states in the metallic particles, which quickly thermalize with particle phonons and thereby increase the surface temperature of the particle. The vibrational lifetime might thus be too short for vibrational energy transfer to be of any relevance in this case. It has been concluded that the cross peaks, which are observed between CO adsorbates on two different binding sites of a nanoparticle, reflect the heating of the latter.^43,44^

Here, we investigate adsorbate-adsorbate as well as substrate-adsorbate interactions on highly heterogeneous platinum layers. In a first step, we show that qualitatively the analogous effect as described above for nanoparticles of controlled size and shape^43,44^ occurs for CO adsorbed to thin films of Pt. In a second step, we extend investigations to an analogous sample system (CN^−^) with significantly longer vibrational lifetime. In contrast to the case of CO adsorbed to Pt layers, the adsorbate-substrate coupling is weaker, and the vibrational energy dissipates mostly into the solvent. Despite its significantly longer lifetime, however, vibrational energy transfer between adsorbed molecules is still not observable. Our results therefore allow us to generalize that adsorbate-adsorbate interactions are insignificant on such highly heterogeneous surfaces.

## II. MATERIALS AND METHODS

2D ATR IR experiments were performed as described in detail before.^16,31^ In brief, the signals are recorded at 5 kHz repetition rate using a pump-probe (PP) 2D IR setup^46^ combined with a single-reflection ATR cell with a CaF_2_ prism as the ATR substrate. Pump and probe pulses were derived from a single optical parametric amplifier^47^ (∼100 fs) and overlapped at the reflecting plane of the ATR prism. Behind the sample cell, the signals are balanced-detected by the use of a 2 × 32 pixel MCT array.

Pt layers were deposited on the CaF_2_ ATR prisms by Ar^+^-ion sputter coating in a Bal-tec SCD 500 sputter coater (Leica Microsystems, Vienna, Austria). Sputtering was carried out at a working distance of 50 mm, a pressure of 0.8 × 10^−5^ mbar, an Ar pressure of 0.1 mbar, and a current of 8 mA. This resulted in a deposition rate of 0.02 nm s^−1^, as determined with a quartz microbalance during the sputtering process. Sub-nanometer thicknesses of the metal layers (0.1–0.3 nm average thickness) were used in this study in order to avoid Fano-type lineshape distortions of the adsorbate vibrational signals.^31,48,49^ Scanning electron microscopy (SEM) on a Zeiss Auriga 40 microscope (Carl Zeiss, Oberkochen, Germany) was used to analyze the sputter-coated surfaces used for 2D ATR IR experiments. Imaging was done using an In-lens secondary electron detector and an acceleration voltage of 5 kV. We note that all samples for SEM analysis had to be coated with an additional 2 nm thin layer of carbon to make the otherwise non-conductive substrates applicable to high-resolution imaging.^50^

Adsorption of the sample molecules was performed by flowing an aqueous solution inside a sample cell mounted on top of the ATR prism, which has either been saturated with an approximately 50%/50% mixture of ^12^C^16^O/^13^C^18^O or contained a total concentration of 5 mM of K^12^C^14^N/K^13^C^15^N, in either case prepared from doubly deionized water. An additional experiment was carried out on a mixture of ^12^C^16^O and ^12^C^14^N^−^ co-adsorbed on the surface, in which case the concentrations have been adjusted such that the 2D ATR IR intensity was approximately equal near zero population time zero (vide infra). Adsorption was carried out until saturation of the surface was achieved (∼2 h), as determined by constant pump-probe signals of the adsorbates measured *in situ* in the 2D ATR IR spectrometer. We furthermore investigated the vibrational dynamics of CO in dependence of surface coverage. Due to the fast and strong adsorption of CO on Pt, surface dilution required to first immerse the Pt-coated ATR prisms in a dilute (1 mM, ethanol) solution of benzenethiol for 1 h followed by a subsequent incubation with a ^12^C^16^O/^13^C^18^O containing solution.^30^ The adsorption of the benzenethiol on Pt blocks surface adsorption sites and therefore results in a larger average distance between CO molecules. For any of these surface preparations, the signals did not change over the course of the experiments (3–4 h), indicating the high stability of the metal layers as well as the negligible desorption of the adsorbates.

**FIG. 1.**
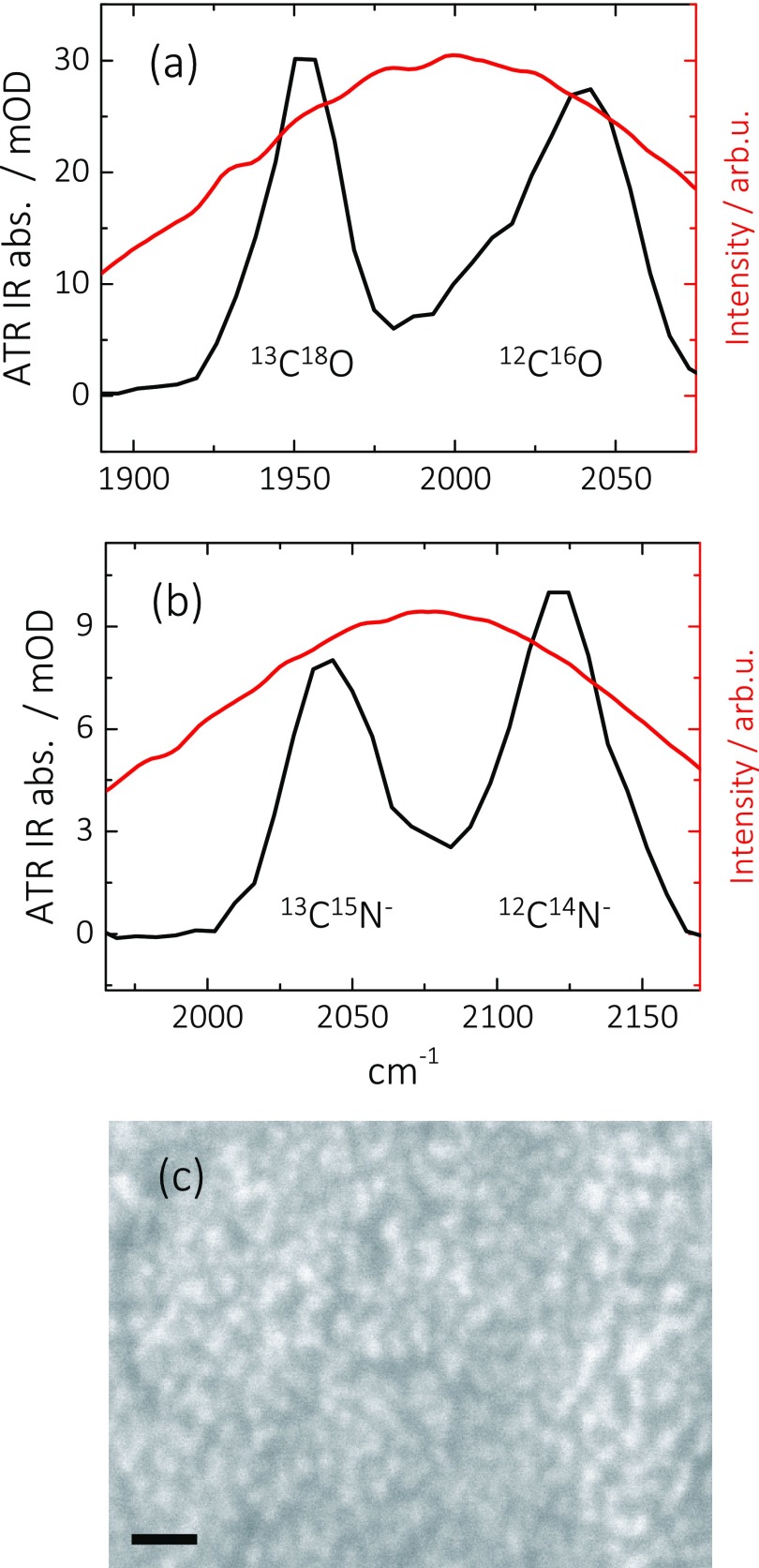
ATR IR absorption spectra of (a) ^12^C^16^O (∼2045 cm^−1^)/^13^C^18^O (∼1955 cm^−1^) and (b) ^12^C^14^N^−^(∼2125 cm^−1^)/^13^C^15^N^−^(∼2040 cm^−1^) on a sputter-coated Pt layer immersed in water. (c) SEM image of a typical Pt layer (0.1 nm) on CaF_2_, consisting of aggregated patches with lateral extensions between 2 and 10 nm, separated by gaps of similar size. The scale bar is 20 nm, and the image pixel size is 323.5 pm.

Figure 1 shows *in situ* measured ATR IR absorption spectra of isotope mixtures of (a) ^12^C^16^O (∼2045 cm^−1^)/^13^C^18^O (∼1950 cm^−1^), and (b) ^12^C^14^N^−^ (∼2125 cm^−1^)/^13^C^15^N^−^ (∼2040 cm^−1^), adsorbed in a linear binding configuration^35^ to thin Pt layers on CaF_2_ ATR substrates (in the case of CO, the frequency of the bridged configuration is outside the spectral window^51–53^ of Figure 1). Both the CO and the CN^−^ isotope mixture results in two bands, separated by about 80–90 cm^−1^ and with the lower frequency band being attributable to the doubly isotope-labelled species. All bands exhibit a width of 30–50 cm^−1^, as is typical for small molecules adsorbed to heterogeneous metal layers prepared by sputter-coating.^31,48^ In the 2D ATR IR experiments, the laser spectra were tuned such that they spectrally covered both bands with approximately the same intensity (red lines in Figures 1(a) and 1(b)). Figure 1(c) shows a scanning electron microscopy (SEM) image of a typical sample. The metal layer consists of randomly structured Pt patches (light regions) with a heterogeneous distribution of dimensions of about 2–10 nm. The patches are only partially connected with voids of similar size (dark regions).

## III. 2D ATR IR RESULTS

### A. CO on Pt

**FIG. 2.**
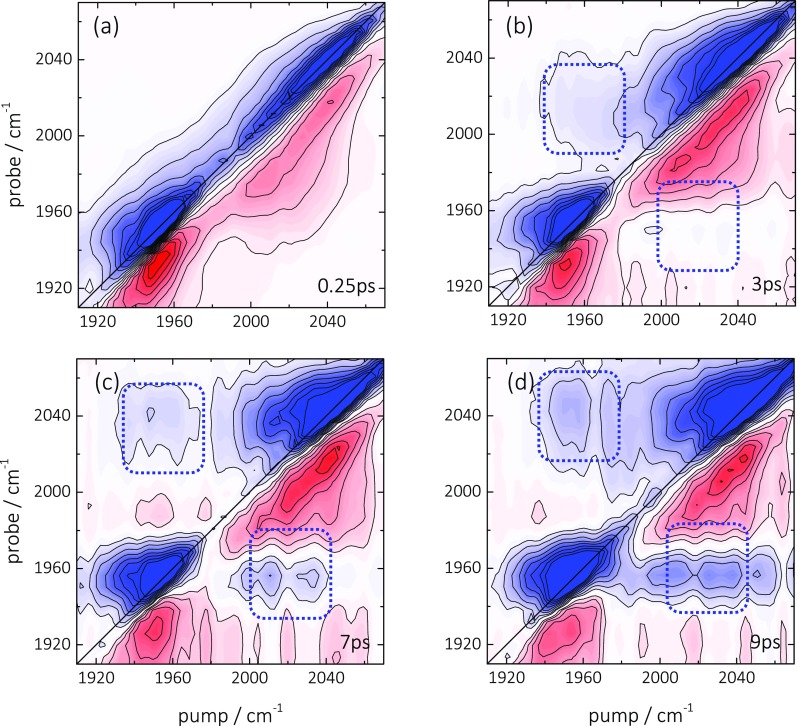
(a)–(d) 2D ATR IR spectra of ^12^C^16^O (∼2045 cm^−1^)/^13^C^18^O (∼1955 cm^−1^) linearly bound to a thin Pt layer (0.1 nm) immersed in water at indicated population waiting times. Blue signals correspond to GSB/SE contributions whereas red signals represent ESA signals. The dashed square indicates the position of the cross-peak between the two CO signals. The GSB/SE diagonal signals are saturated by a factor of two in order to better emphasize the weak cross peaks.

Figures 2(a)–2(d) show 2D ATR IR spectra of the ^12^C^16^O/^13^C^18^O sample at a series of population delays. Initially ((a) T = 0.25 ps) the 2D ATR IR signals consist of pairs of peaks located at the diagonal, which are associated with ground-state bleach/stimulated emission (GSB/SE, blue) and excited-state absorption contributions (ESA, red). These features exhibit strong spectral elongation in the direction of the diagonal, indicative for strong inhomogeneous broadening of the absorption bands, which stems from the heterogeneity of the metal layer.^31,48^ No cross-peaks between the two CO bands are observed at the initial delay time of 0.25 ps. Such an initial cross peak would be expected if the coupling between different molecules would be strong enough to delocalize the vibrational excitation over more than one molecule.^19,31^ That situation could be expected for a very close proximity of the adsorbate molecules on the surface, or even more so when two molecules coordinate to the same Pt atom on a rough surface.^35,36,43,44,54,55^ The missing initial cross peak thus indicates that none of these possibilities correctly describes the situation of CO at the Pt surface. However, even if the coupling is too weak for an instantaneous cross-peak to be observed, vibrational energy transfer could occur as a function of the population time in the 2D ATR IR experiment. In fact, cross-peaks above and below the diagonal grow in on a few picosecond timescale (indicated in Figures 2(b)–2(d) by the blue-dashed squares). Even though an interpretation as vibrational energy transfer is appealing, since it would reveal a measure of the distance between CO molecules on the surface (Eq. (1)), we will demonstrate that a different mechanism is responsible for the appearance of these cross-peaks, that is, the cross-peaks are caused by the heating of the Pt layer due to the rapid energy transfer from the adsorbate to the substrate.

**FIG. 3.**
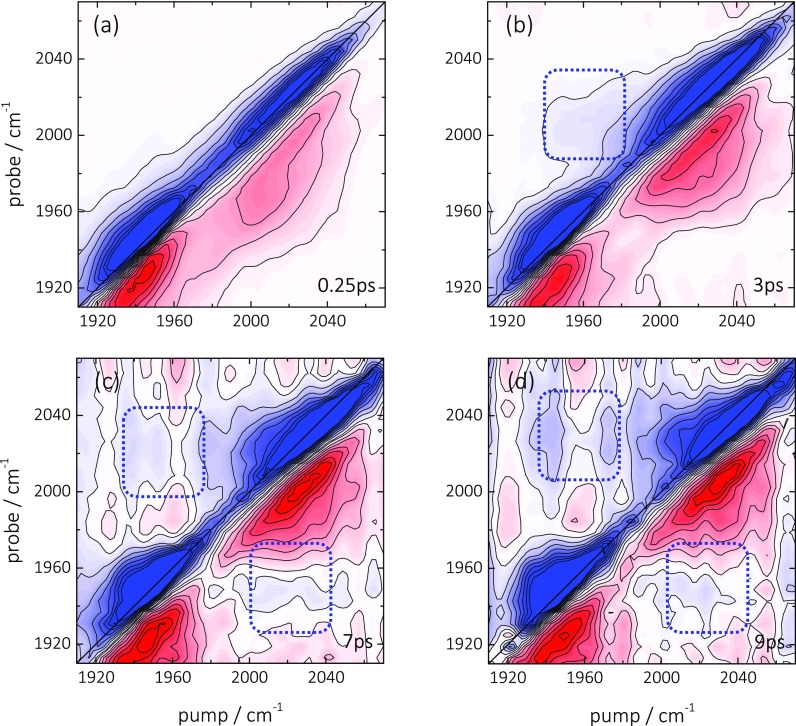
(a)–(d) 2D ATR IR spectra of ^12^C^16^O (2045 cm^−1^)/^13^C^18^O (1955 cm^−1^) with reduced (30%) surface-coverage on a thin Pt layer (0.1 nm) immersed in water at the indicated population waiting times. Blue signals correspond to GSB/SE contributions whereas red signals represent ESA signals. The dashed square indicates the position of the cross-peak between the two CO bands. The GSB/SE diagonal signals are saturated by a factor of two in order to better emphasize the weak cross peaks.

In a first step, we provide compelling evidence with the help of surface dilution experiments that the cross-peaks are not caused by vibrational energy transfer between the CO molecules. Similar to Nuclear Overhauser Enhancement Spectroscopy in nuclear magnetic resonance^56^ or Förster energy transfer in fluorescence spectroscopy,^57^ the vibrational energy transfer rate between a donor and an acceptor molecule scales as

$$k\propto \mu^{4}/r^{6},$$ (1)

where μ is the transition dipole of the CO vibration and r the donor/acceptor distance.^58–61^ Diluting the surface coverage c of CO molecules therefore strongly influences any existing rate of vibrational energy transfer, scaling as $c^{3}$ when assuming that the molecules are randomly distributed on the surface and not clustering. Figure 3 shows 2D ATR IR spectra of ^12^C^16^O/^13^C^18^O obtained from a surface coverage of only ∼30% of the experiments shown in Figure 2, as determined from *in situ* measured ATR IR absorbance spectra. Despite the reduction of the coverage, which should reduce the transfer rate by a factor ∼30, cross-peaks are still clearly apparent (dashed squares) with an intensity measured relative to the diagonal peaks that is similar to the fully saturated surface (Figure 2). This finding evidences that the cross-peaks do not originate from inter-adsorbate vibrational energy transfer along the lines of Eq. (1).

**FIG. 4.**
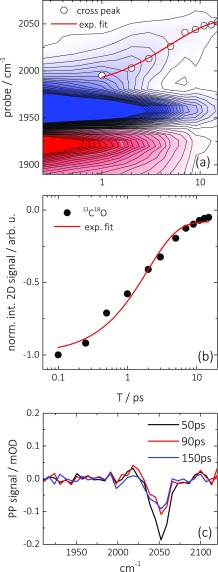
(a) Vibrational dynamics of diagonal and cross-peak signals from 2D ATR IR spectra after integration of a small spectral region of the pump axis (1955 ± 5 cm^−1^), as described in the text. Open circles represent spectral positions of the maximum cross-peak signal at several population delays. The red line indicates an exponential fit to the spectral evolution. The GSB/SE signal is saturated by a factor of two in order to better emphasize the weak cross peaks. (b) Normalized vibrational relaxation dynamics from 2D ATR IR data of ^13^C^18^O (symbols) including an exponential fit (red line). (c) Pump-probe signal at later delay times of only ^12^C^16^O adsorbed on a thin Pt layer immersed in water.

A detailed analysis of the time-dependence of the 2D IR spectra reveals the mechanism that gives rise to the cross peaks. To that end, we plot in Figure 4 the time-dependence of the diagonal and the upper-left cross-peak by integrating the 2D ATR IR spectrum measured at saturated CO coverage (Figure 2) over a small region (∼10 cm^−1^) along the pump axis around the maximum GSB/SE signal (1950–1960 cm^−1^) at a series population delays. This yields the signal shown in Figure 4 with a probe spectral axis and a population delay axis. The intense GSB/SE (1955 cm^−1^) and its associated ESA signal (1920 cm^−1^) correspond to the dynamics of the ^13^C^18^O diagonal peak, while the spectrally evolving contribution from 1 to 15 ps (with significantly lower intensity) corresponds to the cross-peak feature. A single exponential fit of the GSB and ESA diagonal features reveals a decay with a time constants of 1.9 ps (Figure 4(b), red line), which reflects the rapid vibrational relaxation of surface-bound CO, in agreement with Ref. 31. The fit includes a constant offset, which however describes the experimental data at longer delay times (>8 ps) only poorly. At later times, a second process sets it, which is investigated by broadband-pump-IR probe experiments due to the better signal-to-noise ratio (see Figure 4(c), done for a ^12^C^16^O sample). The signal reflects a line-broadening together with a spectral shift, which is typical for thermal effects in ultrafast IR spectroscopy.^62–66^ It decays on a much slower ∼100 ps timescale, which reflects the cooling of the nano-structured thin Pt layer into the CaF_2_ substrate and the solvent.

An interesting feature in Figure 4(a) is the continuous shift of the spectral maximum of the cross-peak feature, which can first be identified with an onset at ∼1 ps, (open circles). An exponential fit to the frequency position (red line) yields a time constant of 5 ps. Adsorbate-substrate energy transfer provides an explanation for the continuous spectral evolution. That is, after pumping the 1950 cm^−1^ band of ^13^C^18^O, rapid vibrational relaxation initially transfers the excess energy to electron-hole pair excitations in the Pt layer. The resulting thermalization leads to a spectral broadening and shift of the absorption bands of both CO species, thereby inducing a cross-peak at the CO band positions. From the spectral shift of this cross-peak, we can determine a time-constant of 5 ps for this thermalization process, which is in good agreement with the earlier reports.^67,68^

The transport of heat should also have a time-dependence, which one might expect to resolve in the dilution experiment of Figure 3. An estimate of the speed, with which vibrational energy is transported by the phononic system of the Pt layer, is given by the speed of sound of Pt (about 3.7 nm/ps).^69^ Energy is locally deposited in the Pt layer by vibrational relaxation of the surface-bound CO with a time constant of 1.9 ps. On that timescale, energy might be distributed over essentially the complete patches of the nano-structured Pt-layer (see Figure 1(c)); hence, vibrational energy transport in the Pt layer is not the rate-limiting step even in the surface-diluted case.

It should be noted that recent reports studied the coupling of CO molecules from different binding configurations on Pt-nanoparticles suspended in solution (“step atop,” “terrace atop” sites vs. “bridge” sites).^43,44^ In contrast, we here investigate the coupling between molecules in the same binding configuration (linearly bound), generating two distinct bands by employing isotope labelling. Up to this point, our results are, however, in complete agreement with the recent findings.^43,44^ In that case, cross-peaks in 2D IR spectra for Pt nanoparticle ≥2 nm have also been attributed to heating of the nanoparticles and not to vibrational energy transfer. One could imagine that the typical distance between molecules is smaller in our case, but apparently not to the extent that vibrational energy transfer can be observed. Concerning the order of magnitude by which the temperature of the substrate is changed upon relaxation of the adsorbed CO, those previous 2D IR experiments on Pt nanoparticles in solution determined temperature changes between 25 and 60 K.^43,44^ As the conditions of these experiments are comparable to the ones employed here, it can be expected that similar temperature changes occur on our heterogeneous Pt layers as well.

### B. CN^−^ on Pt

**FIG. 5.**
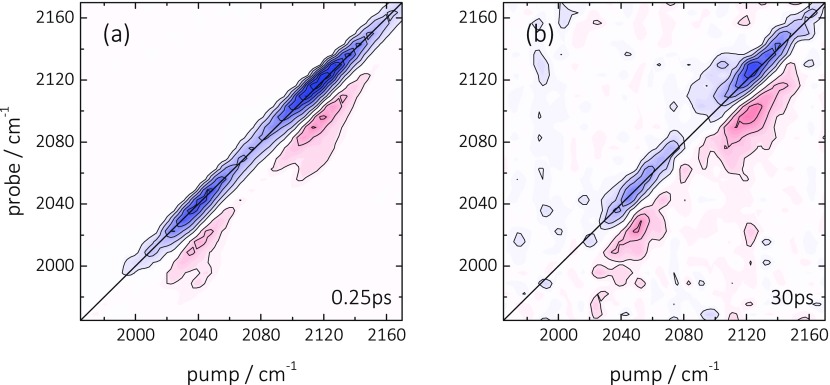
(a) and (b) 2D ATR IR spectra of ^12^C^14^N^−^ (∼2120 cm^−1^)/^13^C^15^N^−^ (∼2040 cm^−1^) adsorbed to a thin Pt layer (0.3 nm) and immersed in water at indicated population delays. No cross-peaks are detectable for ^12^C^14^N^−^/^13^C^15^N^−^ throughout the vibrational lifetime of the adsorbate.

**FIG. 6.**
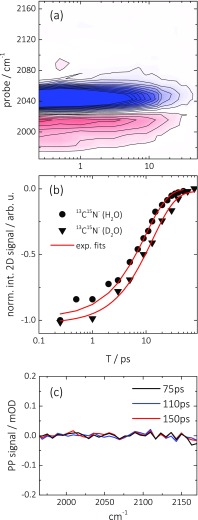
(a) Ultrafast vibrational dynamics of the diagonal signal from the 2D ATR IR spectra of ^12^C^14^N^−^/^13^C^15^N^−^ on Pt (0.3 nm) after integration of a small spectral region of the pump axis (2040 ± 5 cm^−1^). The GSB/SE signal is saturated by a factor of two. (b) Normalized vibrational relaxation dynamics from 2D ATR IR signals of ^13^C^15^N^−^ in contact with water (H_2_O, circles) and heavy water (D_2_O, triangles). Red lines are exponential fits to the experimental data with a decay time of 10 ps in H_2_O and 14 ps in D_2_O. (c) Pump-probe signal at later delay times of only ^12^C^14^N^−^ adsorbed on a thin Pt layer immersed in water.

We now turn to CN^−^ adsorbed to Pt layers, which is a diatomic molecule that is isoelectronic to CO, also easily and strongly binds to Pt, but exhibits a significantly longer vibrational lifetime than Pt-bound CO.^70–72^ Figures 5(a) and 5(b) show 2D ATR IR spectra of a ^12^C^14^N^−^/^13^C^15^N^−^ isotope mixture on a sputter-coated Pt layer. The two spectrally well-separated isotope combinations result in GSB/ESA signals at pump frequencies of 2125 cm^−1^/2040 cm^−1^, respectively. Similar as for CO, the signals appear strongly elongated along the diagonal, reflecting the structural inhomogeneity of the substrate. An integration along the pump axis (∼10 cm^−1^) around the maximum GSB signal at 2040 cm^−1^ for a series of population delays is again used to unravel the vibrational dynamics (Figure 6(a)), revealing a relaxation time of 10 ps (solid circles, Figure 6(b), ^13^C^15^N^−^) without any residual offset for relaxation delays longer than ∼50 ps (Figure 6(c), in contrast to CO, see Figure 4(c)). Vibrational relaxation of CN^−^ on the heterogeneous Pt surfaces is thus significantly slower than for surface-bound CO under the same conditions. In addition, no cross-peak features are observed between the two isotope combinations throughout the vibrational lifetime of the adsorbates, as evidenced by the 2D ATR IR spectra at early (0.25 ps, Figure 5(a)) and late (30 ps, Figure 5(b)) population delays. This indicates that no vibrational energy transfer between the adsorbate molecules can be observed within the investigated temporal range, despite the about 5 times longer vibrational relaxation time as compared to CO, which extends the time window in which vibrational energy transfer could be observed by the same factor. It however also indicates that heating of the substrate does not play any role either in the case of CN^−^, in contrast to CO.

**FIG. 7.**
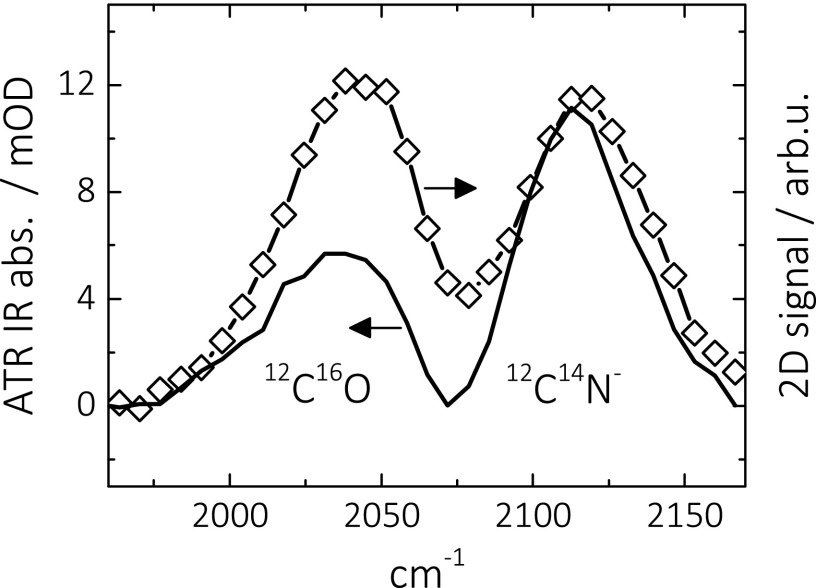
Linear ATR absorption spectrum (solid line, left scale) and diagonal cut through a 2D-ATR IR spectrum (open symbols, right scale, taken at a population time of 0.25 ps) of a mixture of ^12^C^16^O and ^12^C^14^N^−^ co-adsorbed on a thin Pt layer (0.3 nm) immersed in water. The concentrations have been adjusted such that the 2D ATR IR intensity was approximately the same.

Just like it was done for CO in Sec. III A, we will now discuss the CN^−^ results both in terms of vibrational energy transfer and in terms of heating of the Pt surface. Regarding the first possibility, we start with noting that the overall CN^−^ absorption of a saturated Pt surface is about 30% that of CO (Figures 1(a) and 1(b)). That reduced absorption can reflect a lower absorption cross section and/or a lower surface coverage of CN^−^, both entering Eq. (1) in a different way. To disentangle the two effects, we compare in Figure 7 the linear ATR absorption spectrum with a diagonal cut through a 2D ATR IR spectrum of a mixture of ^12^C^16^O and ^12^C^14^N^−^ co-adsorbed on a Pt layer. While the linear ATR absorption spectrum scales as μ^2^, the 2D ATR IR spectrum scales as μ^4^. Comparing both spectra, one can therefore determine the relative absorption cross sections $\mu_{{\text{CN}}^{-}}^{2}/\mu_{\text{CO}}^{2}\approx 0.5$ without having to know the surface coverage of either CO or CN^−^.^50,73,74^ The absorption cross section thus accounts for the reduced overall absorption of CN^−^ (Figure 1(b)) only partially and the remaining factor (0.6) must reflect a lower surface coverage of CN^−^, despite the fact that the surface is saturated under the employed conditions. Combining both factors into Eq. (1), one can estimate that the vibrational transfer rate for CN^−^ should be a factor of about 20 slower than for CO. The factor 5 longer vibrational lifetime of CN^−^ is not able to compensate for that, hence, it is not surprising that we cannot observe any vibrational energy transport either.

**FIG. 8.**
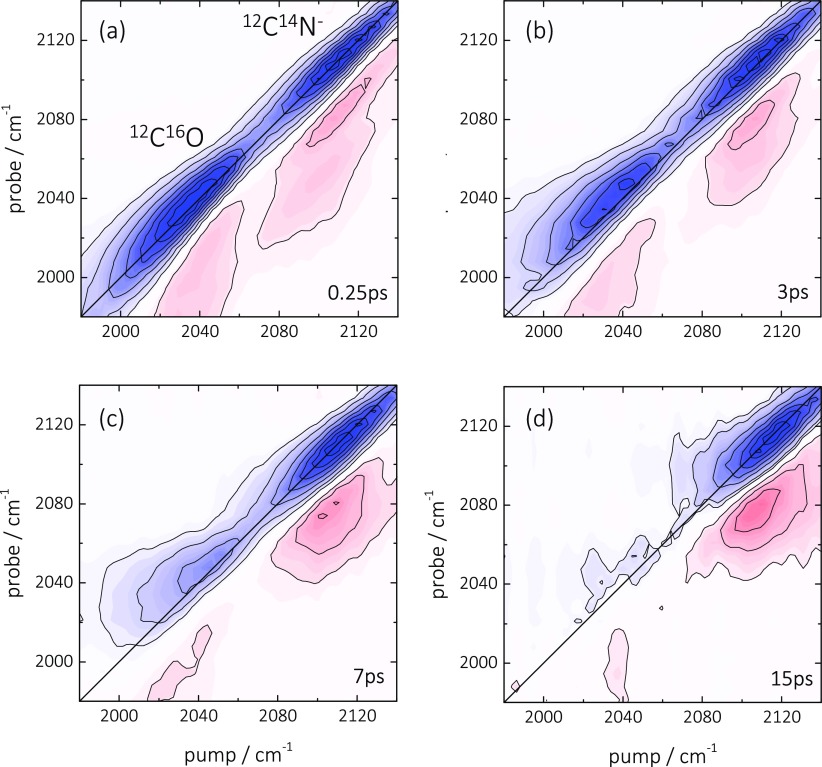
(a)–(d) 2D ATR IR spectra of ^12^C^16^O (CO, 2045 cm^−1^)/^12^C^14^N^−^ (CN^−^, ∼2120 cm^−1^) linearly bound to a thin Pt layer (0.3 nm) immersed in water at indicated population waiting times. Blue signals correspond to GSB/SE contributions whereas red signals represent ESA signals.

To address the missing substrate heating, Figure 8 shows a time-series of the complete 2D ATR IR spectra of the ^12^C^16^O and ^12^C^14^N^−^ mixture. Throughout the vibrational lifetime of CO, no cross-peak features arise between the different adsorbate species. By now no longer surprising, this demonstrates that vibrational energy transfer between both molecules again does not occur. More importantly, it shows that CN^−^ is completely decoupled from the Pt substrate and that in two different aspects. First, the missing CN^−^-pump-CO-probe cross peak (which would be situated below the diagonal) shows that the vibrational energy of CN^−^ is not dissipated into the substrate, since otherwise the generated heat would be sensed by the CO molecule, in analogy to Figure 2. This conclusion is corroborated by the observation that the vibrational relaxation rate depends on the applied solvent and is further slowed down to ∼14 ps in D_2_O (Figure 6(b), solid triangles). A similar effect was already reported for CN^−^ and metal-cyanides^75–77^ in bulk water, as well as for CN^−^ on single crystalline surfaces,^70^ and has been attributed to the lower density of states of D_2_O at the frequency position of the CN^−^ vibration. Hence, the vibrational energy of the CN^−^ is dissipated predominantly into the solvent.

**FIG. 9.**
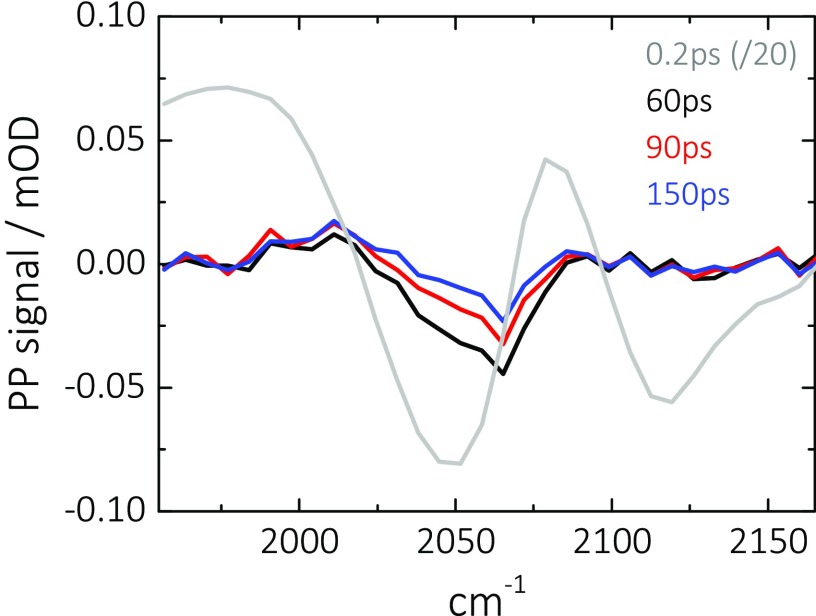
Pump-probe (PP) spectra for co-adsorbed ^12^C^16^O and ^12^C^14^N^−^ on a thin Pt layer (0.3 nm) for the indicated population delays. Spectra for T ≥ 60 ps show partial (only for ^12^C^16^O) heating signals, which exist on longer timescales compared to the vibrational relaxation dynamics.

Second, the missing ^12^C^16^O-pump-^12^C^14^N^−^-probe cross peak (above the diagonal) shows that heating induced by CO is not sensed by the CN^−^ adsorbate. That conclusion is further supported by the pump-probe spectra shown in Figure 9, which are acquired for population delays beyond vibrational relaxation of both adsorbates (>60 ps). In analogy to Figure 4(c), a long-lived thermal signal exists for ^12^C^16^O at about 2060 cm^−1^, which decays on a much slower, few 100 ps time scale reflecting the cooling of the Pt layer on the surface. No such signal is seen for ^12^C^14^N^−^, indicating that the relaxation-induced temperature changes in the substrate, originating from the ^12^C^16^O adsorbate, does not result in any broadening or spectral shift of the ^12^C^14^N^−^ absorption band.

## IV. DISCUSSION AND CONCLUSIONS

Taking together the results from CO and CN^−^ on a Pt layer, we conclude that the absence of inter-adsorbate energy transfer in the case of surface-bound CO is not caused by a competing rapid vibrational relaxation rate (2 ps); the missing vibrational energy-transfer on the picosecond timescale appears to be a rather general observation for small molecules on heterogeneous metal surfaces, even with significantly longer vibrational lifetimes, as in the case of CN^−^. That conclusion is astonishing in different ways. First, small molecules adsorbed in a linear binding configuration at a surface exhibit minimal intra-molecular conformational heterogeneity and ideal relative orientation of their transition dipoles to couple. Second, metal-carbonyls and metal-cyanides exhibit about the strongest transition dipoles known in the IR spectral range.^78–80^ The coupling strength and energy transfer rate strongly depends on the magnitudes of the transition dipoles (Eq. (1)) as well as their relative orientation, both points should thus promote the observation of energy transfer at interfaces.^19,81^ However, another factor determining the strength of inter-molecular interactions is the distance between the respective oscillators. Typically, functional groups that show vibrational energy transfer exhibit inter-molecular distances of much less than a nanometer.^44,81–83^ It is therefore possible that even in the case of a saturated surface-coverage, as applied here, the distance between the adsorbate molecules is too large to result in energy transfer at the interface. Taking the lateral dimensions of the Pt patches of the layers in our experiment (2–10 nm), it is thus likely that only a few molecules adsorb on every particle.

In various earlier studies on 1D vibrational spectroscopy of adsorbates on metal surfaces, frequency shifts and absorbance variations in dependence of surface coverage have been interpreted in terms of dipole-dipole couplings.^35–39^ The 2D ATR IR experiments reported here, however, clearly show that the adsorbate molecules do not interact with each other. Our results thus challenge the previously developed interpretation and require a different interpretation for these experimental observations. In this regard, a promising starting point in this direction will be to compare results from different surface morphologies such as nanostructured versus single crystalline surfaces.

Also surprising is the fact that CO and CN^−^ couple differently to the Pt substrate, despite the fact that they are isoelectronic. We currently have no explanation for this observation, and more experiments, possibly involving a systematic screening of different Pt-bound molecules and ions, will be necessary to clarify that point.

We finally discuss the missing interactions between adsorbates at heterogeneous surfaces in more general terms. In this context, it is important to note that the dynamics of small molecules at solid-liquid/gas interfaces play a prominent role in for instance heterogeneous catalysis.^3,18,84,85^ Moreover, many systems for heterogeneous catalysis exploit the high surface area and size-related physical and chemical properties of nano-structured surfaces.^85–88^ The properties and dynamics for molecules at surfaces can be controlled by three factors, i.e., (i) substrate-adsorbate, (ii) adsorbate-adsorbate, or (iii) adsorbate-bulk environment interactions. From these three contributions, the results presented here indicate the importance of substrate-adsorbate interactions, whereas recent reports^31,48^ have already highlighted the importance of existing adsorbate-bulk environment interactions, for instance via hydrogen-bonding to solvent molecules. Adsorbate-adsorbate interactions in the form of non-resonant vibrational energy transfer, on the other hand, seem to play only a very minor role for the adsorbate's vibrational dynamics. We note, however, that resonant energy transfer between adsorbates might be faster due to spectral overlap between the donor and acceptor spectral bands. It will be important to investigate in future studies, whether the observation of adsorbate-adsorbate interactions indeed requires at least partially spectrally overlapping bands, chemical interactions such as hydrogen-bonding or complexation, and if non-chemical interactions such as dipole-dipole coupling or resonant energy transfer can have any influence on heterogeneous catalysis.

In conclusion, we have investigated ultrafast vibrational dynamics of carbon monoxide and cyanide adsorbed at the metal-water interface of sputter-coated thin Platinum (Pt) layers with 2D ATR IR spectroscopy. Isotope-labelling was employed to study possible interactions of the adsorbate molecules and substrate-adsorbate energy transfer. Rapid (2 ps) and slower (10 ps) vibrational relaxation was observed for CO and CN^−^, respectively, which originates from present/absent vibrational energy transfer from the adsorbate to the substrate. The excess vibrational energy of the adsorbates transferred to the metal layer excites electron-hole pairs in the conduction band of the metal, the thermalization of which to particle phonons results in heat-induced cross-peaks in the 2D ATR IR spectra, only in case of CO. Inter-adsorbate vibrational energy transfer or vibrational coupling could not be detected throughout the vibrational lifetime of the adsorbates. The missing interactions on the picosecond timescale suggest that even in the case of surface coverages close to saturation, the typical inter-molecular distance between adsorbate molecules is relatively large. We therefore postulate that inter-adsorbate energy dissipation is probably not very relevant for instance for catalytic reactions at heterogeneous surfaces.
